# Human in vitro neuromuscular junction model to functionally dissect the pathogenic mechanism of anti-AChR autoantibody-positive myasthenia gravis

**DOI:** 10.1186/s40360-025-01056-1

**Published:** 2025-12-12

**Authors:** Baehyun Shin, Monica Wang, John Yim, Elisa Kwon, Margaret H. Magdesian, Camil E. Sayegh, Jason E. Ekert, Douangsone D. Vadysirisack

**Affiliations:** 1 UCB, 87 Cambridgepark Dr, Cambridge, MA 02140 USA; 2 Novo Nordisk, 300 North Beacon Street, Watertown, MA 02135 USA; 3 Boston Children’s Hospital, 300 Longwood Ave, Boston, MA 02115 USA; 4 Ananda Devices Inc., #135-500 Boulevard Cartier Ouest, Laval, QC H7V 5B7 Canada; 5 Psivant Therapeutics, 451 D Street, Boston, MA 02210 USA; 6 Hillstar Bio, Boston, MA USA

**Keywords:** Myasthenia gravis, Neuromuscular junction model, Autoantibodies, Complement activation, Zilucoplan, Microfluidic platform

## Abstract

**Background:**

Myasthenia gravis is a rare autoimmune disease mediated by autoantibodies directed against acetylcholine receptors (AChRs) at the neuromuscular junction. These autoantibodies cause dysfunction through AChR blockade, AChR degradation due to crosslinking and internalisation, and complement activation.

**Methods:**

A novel in vitro model of the human neuromuscular junction was established on a microfluidic platform to investigate the effect of anti-AChR autoantibodies on complement activation and neuromuscular transmission and the mechanism of action of complement inhibition in myasthenia gravis. The NeuroMuscle^TM^ platform enabled the connection of human induced pluripotent stem-cell-derived motor neuron spheroids with three-dimensional cultures of skeletal muscle fibres, forming functional neuromuscular junctions. Functional connectivity was assessed by glutamate stimulation of motor neuron spheroids and monitoring of calcium transients in genetically encoded calcium indicator protein 6 (GCaMP6)-transduced muscle fibres.

**Results:**

Incubation of in vitro neuromuscular junction tissues with sera from patients with anti-AChR autoantibody-positive myasthenia gravis, in contrast to healthy controls, induced a significant increase in membrane attack complex (MAC) deposition and complement split products, accompanied by a notable reduction in calcium transients. Treatment with zilucoplan, a complement component 5 (C5) inhibitor, prevented complement activation and preserved neuromuscular junction functional integrity. The model demonstrated that complement-mediated damage is a major driver of neuromuscular junction functional impairment in the myasthenia gravis patient sera tested in this study. Furthermore, the study explored the reversibility of neuromuscular junction damage, revealing that shortening the delay before initiating complement inhibitor treatment in the in vitro neuromuscular junction tissues enhances the reversibility of neuromuscular transmission.

**Conclusions:**

These findings offer a mechanistic rationale for the observed clinical response in patients with anti-AChR autoantibody-positive myasthenia gravis treated with C5 inhibitors. The in vitro neuromuscular junction model provides a robust platform for studying the mechanistic pathways of complement-mediated damage and evaluating therapeutic interventions for myasthenia gravis.

**Supplementary information:**

The online version contains supplementary material available at 10.1186/s40360-025-01056-1.

## Background

The neuromuscular junction (NMJ) is a specialised synapse that mediates the communication between motor neurons (MNs) and skeletal muscle fibres, enabling voluntary movement and muscle contraction. The development and maintenance of the NMJ depend on the coordinated interactions of multiple cellular and molecular factors, including agrin, laminin, neuregulins, acetylcholine receptors (AChRs), and muscle-specific tyrosine kinase [1]. Dysfunction or degeneration of the NMJ can lead to various neuromuscular disorders such as myasthenia gravis (MG) [2].

MG is a chronic, autoimmune neuromuscular disease characterised by varying degrees of weakness of the skeletal muscles [3, 4]. This disease is caused by a breakdown in the normal communication between nerves and muscles due to autoantibodies that target proteins essential for neuromuscular transmission [5]. One of the most targeted components is the nicotinic AChR located at the NMJ. In MG, disruption of receptor functions through mechanisms such as blocking of the acetylcholine binding site, accelerating the degradation of the receptors, modulating the expression of the receptors, and activating complement causes damage to the NMJ [6, 7]. Complement activation, in particular, plays a central role in this process, with anti-AChR antibodies triggering the classical complement cascade and leading to MAC formation at the postsynaptic membrane – this results in structural damage and impaired neuromuscular transmission, as supported by both historical and recent studies showing MAC deposition and elevated complement split products in MG patients [8–12].

Treatments for MG have evolved with the development of new targeted therapies, including those that modulate the complement system. Eculizumab, a monoclonal antibody that inhibits complement C5, was the first complement inhibitor approved for generalised MG and has demonstrated significant clinical benefit in refractory MG patients. Ravulizumab is a long-acting C5 inhibitor, with extended dosing intervals, and has shown rapid and sustained improvements in MG symptoms in clinical trials [13]. Zilucoplan, a 15-amino acid macrocyclic peptide inhibitor of human complement C5, [14, 15] is self-administered subcutaneously and has a dual mechanism of action, blocking C5 cleavage and preventing assembly and activity of the C5b6 complex, which distinguishes it from monoclonal antibodies [16]. Zilucoplan has been shown to demonstrate greater permeability than a monoclonal anti-C5 antibody in a reconstituted basement membrane model [16]. Its efficacy has been demonstrated in both a Phase 2 (NCT04115293) and a Phase 3 (RAISE, NCT04277485) clinical trial, showing significant and clinically meaningful improvements in MG symptoms. In addition to complement inhibitors, therapies targeting the neonatal fragment crystallisable receptor, such as efgartigimod and rozanolixizumab, have emerged as effective options by reducing pathogenic immunoglobulin G levels and improving neuromuscular function [17, 18]. These advances reflect a growing therapeutic landscape and underscore the need for mechanistic models to evaluate and compare these interventions.

Several in vitro NMJ models have been established using animal or human cells which could be used to investigate the effect of complement activation and inhibition on the NMJ in MG. However, most of these models are based on two-dimensional cultures that fail to recapitulate the complex three-dimensional (3D) architecture and physiology of the NMJ in vivo [19–21]. Moreover, the availability and accessibility of human primary cells, especially MNs, are limited and pose ethical challenges. However, recent advances in stem cell technology and tissue engineering have opened new avenues for generating human in vitro NMJ models. Pluripotent stem cells, such as embryonic stem cells and induced pluripotent stem cells (iPSCs), can be differentiated into various cell types of the NMJ, including MNs, skeletal muscle progenitors, and glial cells [22–24]. Furthermore, 3D culture methods, such as hydrogels, scaffolds, and microfluidics, can provide more biomimetic and physiologically relevant environments for NMJ formation and function [25–27]. A relevant NMJ model of MG should mimic the disease by reproducing the reduction of functional nicotinic AChRs at the NMJ, decreased NMJ stability, complement activation, and blocking of neuromuscular signal transmission [27–29]. Therefore, the development of an appropriate in vitro NMJ model for MG provides an opportunity to functionally dissect anti-AChR-mediated pathogenic mechanisms in MG.

In this study, we developed a novel co-culture system using human iPSC-derived MNs and skeletal muscle fibres within a microfluidic device that enables the functional assembly of a 3D NMJ. Using the model, we aimed to characterise the morphological and functional maturation of the NMJ and demonstrate its applicability for MG disease modelling. Additionally, we sought to explore how complement C5 inhibitors, such as zilucoplan, may work in vivo and provide insights into the observed therapeutic efficacy in clinical trials.

## Methods

### Microfluidic chambers

The microfluidic chambers’ fabrication followed a similar process to that previously reported [30]. Briefly, the masters for the microfluidic chambers were manufactured in the McGill Nanotools Microfab (McGill University, Montreal, QC, Canada), and the chambers were prepared with polydimethylsiloxane using the Sylgard 184 Silicone elastomer kit (Dow Corning, Midland, MI, USA). The polydimethylsiloxane patterns were cut to size and assembled on 35-mm glass-bottom dishes (MatTek, Ashland, MA, USA) using plasma bonding methods, according to the instructions of the plasma machine (Glow Plasma System) manufacturer (Glow Research, Tempe, AZ, USA).

### Differentiation of human iPSC into motor neuron spheroids

All cell cultures were maintained at 37 °C, 5% CO_2_. Human iPSCs ASE-9211 (Applied StemCell, Inc., Milpitas, CA, USA) were cultured for 2–3 weeks on Matrigel^®^ hESC-qualified matrix (Corning) in mTeSR™ Plus medium (STEMCELL Technologies, Waterbeach, Cambridge, UK) prior to passaging for differentiation. Differentiation into MNs was achieved using an optimised 2-week protocol (Supplementary Figure 1) previously described.[31]

Briefly, iPSCs were dissociated into single cells using Gentle Cell Dissociation Solution (STEMCELL Technologies) and plated at a density of 1 × 10^6^ cells per well of an AggreWell™800 24-well plate (STEMCELL Technologies) in supplemented neurulation medium (a 1:1 mixture of Neurobasal and Advanced DMEM/F12) for 24 hours to initiate the formation of neurospheres. The cells were kept in culture for 10 days with medium exchanges three times a week using the medium formulations previously described in the literature for optimised MN differentiation [31]. On Day 11, neurospheres were filtered using a 37 µM reverse strainer and transferred to a 6-well plate with maturation medium A [a 1:1 mixture of Neurobasal (Thermo Fisher Scientific) and Advanced DMEM/F12 (Thermo Fisher Scientific) supplemented with 1% N2 (Thermo Fisher Scientific), 2% B27 (Thermo Fisher Scientific), 2 mM GlutaMAX (Thermo Fisher Scientific), 0.1 mM 2-mercaptoethanol (Sigma-Aldrich), 0.5 µM ascorbic acid (Sigma-Aldrich), and 100 µg/mL brain-derived neurotrophic factor (R&D Systems), and glial cell-derived neurotrophic factor (R&D Systems)]. On Day 14, the medium was replaced with MN maturation medium B (same as medium A supplemented with MEM nonessential amino acids [Thermo Fisher Scientific], 0.025 mM L-glutamic acid [Sigma-Aldrich]). A 75% media change was performed every other day.

### Formation of 3D neuromuscular tissues in NMJ microfluidic device

**Fig. 1:**
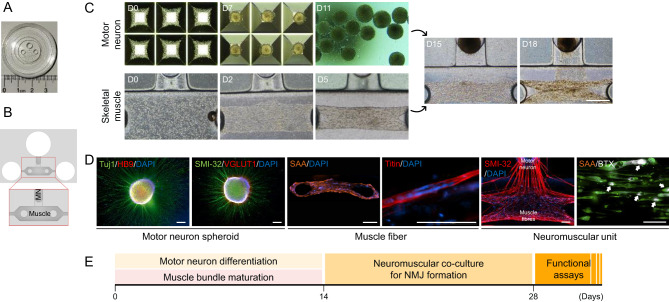
3D compartmentalised design of human neuromuscular tissues on a microfluidic device. (**A**) The fabricated NeuroMuscle^TM^ device uses the PDMS inlet microchannel to form a single NMJ microfluidic device, each composed of a muscle compartment with muscle fibre attaching pillar structures and the MN spheroids compartment. The muscle compartment has two medium reservoirs on each side. The MN compartment has a single medium reservoir. (**B**) Photo of the 3D NeuroMuscle^TM^ microstructure. (**C**) Human iPSCs were seeded on an AggreWell™800 plate at Day 0 and MN spheroids were generated at Day 7. GCaMP6-transduced human skeletal myoblasts were injected into the muscle compartment of the NeuroMuscle^TM^ device at day 0. Spontaneous self-organisation of myoblast into muscle bundle was observed after Day 5. A MN spheroid was introduced into the neuronal compartment and embedded in collagen gel. Thin neurite outgrowth was observed at Day 15 and many thick nerve bundles reached the muscle tissue at day 18, resulting in the formation of NMJs. Scale bars, 500 µm. (**D**) Differentiation of MN spheroids were characterised by the immunostaining of Tuj1, HB9, SMI-32, and VGLUT1. The pattern of a sarcomere structure stained by sarcomeric α-actinin and titin. SMI-32 staining showed that neurites from the motor neuron innervated muscle cells. AChR clusters are labelled with α-bungarotoxin (BTX; monochrome) and indicated by white arrows. Scale bars, 200 µm. (**E**) Schematic illustration of the differentiation and co-culture of the MN spheroid and muscle cells in a NeuroMuscle^TM^ device. 3D, three-dimensional; AChR, acetylcholine receptor; BTX, α-bungarotoxin; D, day; DAPI, 4’,6-diamidino-2-phenylindole; GCaMP6, genetically encoded calcium indicator protein 6; iPSC, induced pluripotent stem cell; MN, motor neuron; NMJ, neuromuscular junction; PDMS, polydimethylsiloxane; SAA, serum amyloid a; Tuj1, class III beta-tubulin; VGLUT1, vesicular glutamate transporter 1

A fresh vial of primary human skeletal myoblasts (Thermo Fisher Scientific) was thawed in a 37 °C water bath and transduced with lentiviral particles at a multiplicity of infection of 20 to express GCaMP6, a fluorescent calcium indicator LV-CAG-GCaMP6s (SignaGen, Frederick, MD, USA). 1.2 × 10^4^ human skeletal myoblasts were injected into the muscle compartment of the NeuroMuscle^TM^ microfluidic device (Ananda Devices Inc.) with Ham’s F12 nutrient mixture, 80% collagen cell matrix type 1-A, reconstitution buffer (Collagen Gel Culturing Kit; Wako Chemicals, Richmond, VA, USA) and 10% Matrigel^®^ growth factor reduced basement membrane matrix (Corning) via the right injection port (Fig. 1A). The device was incubated for 5 minutes at 37 °C with 5% CO_2_ to enable the polymerisation. The muscle tissue was then cultured and differentiated as previously described [25] using medium supplemented with horse serum (Thermo Fisher Scientific) and human recombinant IGF-1 (R&D Systems). A 3D skeletal muscle tissue was formed within 24 hours, with both sides of the muscle bundle attached to the pillar structures. After 2 weeks of muscle tissue incubation, fully differentiated and maturated MN spheroids were introduced into the neuronal compartment of the NeuroMuscle^TM^ device (Fig. 1B) with a mixture of Ham’s F12, 80% collagen cell matrix type 1-A and reconstitution buffer (Collagen Gel Culturing Kit; Wako Chemicals). The device was incubated for 5 minutes in the 37 °C incubator to enable the polymerisation before pouring medium into the reservoir of the neuronal compartment. To induce neurite outgrowth and NMJ formation, MN spheroids and 3D skeletal muscle tissues were co-cultured with MN maturation medium A for an additional 14 days.

### Calcium transient analysis

Functional connectivity was assessed with L-glutamate stimulation of MN spheroids and subsequent calcium transients in GCaMP6-transduced muscle fibres. L-glutamate (Abcam) was reconstituted to 0.5 M in 1 N NaOH and then further diluted in neurobasal media without phenol red to 50 µM working concentration. Culture media was removed from the NMJ microfluidic device prior to glutamate injection. 3D skeletal muscle tissues expressing GCaMP6 were imaged using a Cytation 5 cell imaging multimode reader (BioTek, Santa Clara, CA, USA) equipped with reagent injector modules to collect real-time calcium influx after glutamate stimulation. Time-lapse videos were recorded for 20 seconds before stimulation (resting) and an additional 120 seconds after stimulation (induced) under physiological conditions using Gen5 software. Change in fluorescence intensity relative to the resting fluorescence intensity was used to measure the level of neuromuscular transmission (ΔF/F).

To assess the effect of nicotinic AChR antagonist treatment on neuromuscular transmission, NMJ tissues were incubated with 1 µM α-bungarotoxin (BTX) (Thermo Fisher Scientific) and 25 µM d-tubocurarine (DTC) (Sigma-Aldrich, Burlington, MA, USA) for 10 minutes before calcium transient analysis.

To evaluate the impact of MG patient sera on neuromuscular transmission, GCaMP6 fluorescent intensity was recorded prior to serum incubation as a baseline and then captured again after 24 hours of patient serum incubation. GCaMP6 fluorescent intensities from different imaging timepoints were normalised by corresponding baseline intensities.

### MG patient sera and zilucoplan treatment

Sera from five patients diagnosed with MG and confirmed positive for anti-AChR antibodies (Table 1) were added to the NMJ tissue culture media at 35% final concentration, and 5% normal human serum (Complement Technology, Tyler, TX, USA) was supplemented. All MG patient sera were obtained from commercial biorepositories (Discovery Life Sciences and BIOIVT), which confirmed that samples were collected and stored under conditions suitable for complement analysis, in alignment with best practices such as those outlined by Mohebnasab et al [32]. Pooled sera from healthy volunteers (Complement Technology) were used as a control.

**Table 1:** Demographic details of the five MG patients

Patient Number	Gender	Age (years)	Race	Ethnicity	Date of Symptom Onset	Date of Diagnosis	Date of Collection	Method of Diagnosis	AChR Antibody Concentration (nmol/L)	Surgical History	Previous Treatments
MG-1	Female	85	White	Non-Hispanic/ Latino	Unknown	Unknown	1 Oct 2019	Blood test	9.1	N/A	N/A
MG-2	Female	70	Unknown	Unknown	Unknown	Unknown	1 Dec 2018	Blood test	56.9	N/A	N/A
MG-3	Female	29	Caucasian	Hispanic	1 Aug 2014	11 Dec 2019	6 Nov 2020	EMG, blood test	10.4	Cholecystectomy	Pyridostigmine 60 mg
MG-4	Female	59	Caucasian	European	Unknown	10 Jul 2018	16 Jun 2021	Blood test	0.15	Tonsillectomy, tubal ligation, Bartholin’s cyst removal	Pyridostigmine 60 mg, Synthroid^®^ 112 mcg
MG-5	Male	70	White or Caucasian	Non-Hispanic/ Latino	1 Jun 2008	1 Jun 2015	19 Oct 2021	Blood test	0.7	N/A	Azathioprine 50 mg, pyridostigmine 60 mg

AChR, acetylcholine receptor; EMG, electromyography; MG, myasthenia gravis; N/A, not applicable

Zilucoplan (provided by UCB, Brussels, Belgium) was dissolved in dimethyl sulfoxide (DMSO) to prepare a 10 mM stock solution and diluted in culture medium to a final concentration of 1 µM. The final DMSO concentration did not exceed 0.1% (v/v) in any condition. DMSO was used as a vehicle control in all experiments involving zilucoplan.

To ensure objectivity and data integrity, all experiments involving MG and healthy control sera including calcium transient analysis, immunocytochemical staining for MAC deposition, and biochemical quantification of complement split products were conducted in a blinded manner. Sample identities were masked during data acquisition and analysis.

### Immunocytochemical staining

Engineered human skeletal muscle tissues from the NMJ chips were fixed in 4% paraformaldehyde for 30 minutes at room temperature (RT), followed by permeabilisation with 1% (v/v) Triton X-100/dH2O for 20 minutes at RT. The samples were then rinsed twice with phosphate-buffered saline (PBS) and blocked with a blocking buffer (2% [v/v] normal donkey serum and 1% bovine serum albumin in 0.01% [w/v] Triton X-100/PBS) at RT for 1 hour. Subsequently, the samples were incubated overnight with primary antibodies against class III beta-tubulin (1:200; Abcam, Cambridge, UK), HB9 (1:50; Santa Cruz, Dallas, TX, USA), SMI-32 (1:100; BioLegend, San Diego, CA, USA), vesicular glutamate transporter 1 (VGLUT1) (1:100; Abcam), sarcomeric α-actinin (1:100; Abcam), titin (1:200; Developmental Studies Hybridoma Bank, Iowa City, IA, USA), and C5b9 (1:500; BD Biosciences, Franklin Lakes, NJ, USA) in the blocking buffer. The samples were then stained with secondary antibodies conjugated to Alexa Fluor 647 or 488 (donkey anti-mouse or donkey anti-rabbit, 1:200; Thermo Fisher Scientific) in the blocking buffer for 1 hour. Mounting media containing 4′,6-diamidino-2-phenylindole was used to label nuclei. Images were acquired using a Cytation 5 (Agilent, Waldbronn, Germany) equipped with a digital camera. Image quantification of MAC deposition was performed by capturing deposition in five different images.

### Biochemical analysis of complement split products

The concentrations of C5a and sC5b9 (soluble MAC) in the supernatants from NMJ tissues incubated with MG patient sera were measured using the MicroVue™ Complement Multiplex Array (Quidel Corporation, San Diego, CA, USA). The supernatants were diluted with specimen diluent to reach a final serum concentration of 0.1%, ensuring that measurements fell between the lower limit of quantitation and the upper limit of quantitation of each assay. To maintain objectivity and data integrity, the experiment was conducted as a blinded study.

### Statistical analysis

All statistical analyses were performed using GraphPad Prism, Version 9.0 (GraphPad Software, San Diego, CA, USA). Data are presented as mean ± standard error of the mean unless otherwise stated. Comparisons between multiple groups were made using ordinary one-way analysis of variance (ANOVA) for a single variable or two-way ANOVA for the interaction between two independent variables, followed by Tukey’s post hoc test. For comparisons involving only two groups, an unpaired Student’s t-test was used. A p-value of less than 0.05 was considered statistically significant. The lack of a significance symbol indicates that there were no significant differences.

## Results

### Fabrication of a 3D neuromuscular model in microfluidic devices

A microfluidic platform (NeuroMuscle^TM^, Fig. 1A and B) was employed to connect 3D cultures of human primary skeletal muscle fibres and MN spheroids derived from human iPSCs to form functional NMJs. Primary human skeletal myoblasts were injected into the muscle compartment of NeuroMuscle^TM^ with a mixture of collagen and Matrigel^®^. The cells spontaneously formed 3D skeletal muscle fibres around the two post structures (Fig. 1C). After 14 days, the skeletal muscle exhibited regular well-patterned sarcomeric structures along with mature myotube differentiation (Fig. 1D). Immunostaining of sarcomeric α-actinin and titin indicated the maturation of the elongated MNs and mature 3D muscle fibres in the co-culture condition.

A protocol previously described in the literature to accelerate differentiation of human iPSCs into MN [31] was adapted to accelerate MN differentiation (Fig. 1C) and formation of 3D spheroid cultures. Briefly, MN spheroids were formed using a sequential application of several growth factors as described in ‘Methods’ (Supplementary Figure 1). By Day 14, MN spheroids strongly expressed HB9, neurofilament heavy chain (SMI-32), class III beta-tubulin, microtubule-associated protein 2 and VGLUT1, which are mature MN markers (Fig. 1D and Supplementary Figure 2).

On Day 14, fully differentiated MN spheroids were injected into the neuronal compartment of the NeuroMuscle^TM^ (Fig. 1E). Motor neurite elongation in the collagen gel toward the muscle fibre bundle was observed by bright field analysis in 24 hours (Fig. 1C). After 4 days of co-culture, bright field analysis showed many neurites had reached the 3D skeletal muscle fibre (Fig. 1C). Furthermore, thick neurite bundles were observed close to the muscle tissue by 14 days in co-culture (Fig. 1D). Immunostaining of SMI-32 indicated the maturation of the elongated MNs in the co-culture condition (Fig. 1D). AChR clustering was also observed in our 3D muscle fibre reflecting structural innervation and maturation of NMJs (Fig. 1D).

### 3D neuromuscular tissues are functionally innervated

**Fig. 2:**
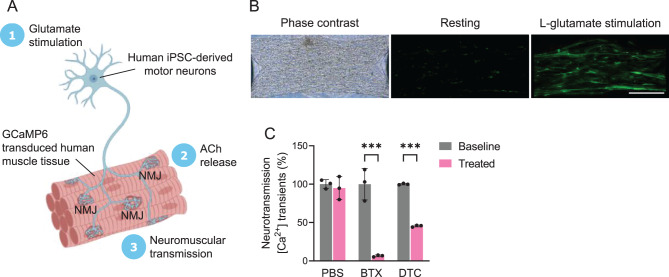
Functional connectivity between motor neuron endplates and muscle fibres. (**A**) NMJ model illustrated with BioRender. Neuromuscular transmission was assessed with L-glutamate stimulation (50 µM) of motor neurons and subsequent calcium transients in GCaMP6-expressing skeletal muscle tissues. (**B**) Phase contrast (left panel) and GCaMP6 fluorescence images of a 3D NMJ co-culture before (middle panel) and after (right panel) treatment with L-glutamate. Scale bars, 500 µm. (**C**) Functional connectivity of the co-cultures was confirmed with glutamate stimulation, which could be blocked by AChR antagonists such as 1 µM α-bungarotoxin (BTX) and 25 µM d-tubocurarine (DTC). Fluorescence intensity was shown as mean ± sd. *n* = 3. ^***^p < 0.001. Statistical significance was analysed by ANOVA (Bonferroni test) using GraphPad software. 3D, three-dimensional; ACh, acetylcholine; AChR, acetylcholine receptor; ANOVA, analysis of variance; BTX, α-bungarotoxin; DTC, d-tubocurarine; GCaMP6, genetically encoded calcium indicator protein 6; iPSC, induced pluripotent stem cell; NMJ, neuromuscular junction; PBS, phosphate-buffered saline; sd, standard deviation

To assess the functional connectivity of MN spheroids and 3D skeletal muscle tissue, we employed L-glutamate, an excitatory neurotransmitter that selectively stimulates MN spheroids without affecting muscle tissues. Human skeletal myoblasts were transduced with the GCaMP6 to monitor the subsequent calcium transients in muscle tissues (Fig. 2A). The neuromuscular transmission was observed on Day 14 of co-culture (Fig. 2B), signifying the formation of functional connectivity between MN endplates and skeletal muscle tissues. To further confirm that the presynaptic activation of MN spheroids induced the observed changes in muscle fibre calcium transients, we investigated the effects of postsynaptic blockers, BTX and DTC in the NMJ system. The presence of BTX and DTC, both nicotinic AChR antagonists, significantly inhibited calcium transients (Fig. 2C). This inhibition indicated that neuromuscular transmission was triggered by MN activity rather than spontaneous muscle fibre movement.

### Sera from MG patients induced complement activation and NMJ impairment

**Fig. 3:**
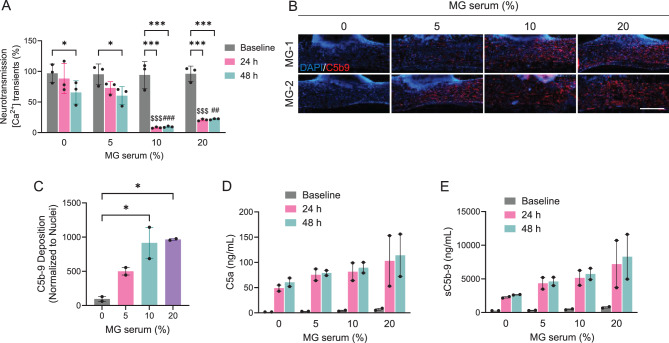
Anti-AChR antibody-positive MG-serum-induced complement activation and impaired neurotransmission (**A**) Incubation of the NMJ model with MG-2 patient sera containing anti-AChR antibody resulted in reduced neuromuscular transmission in a concentration-dependent manner. Fluorescence intensity was shown as mean ± SEM. Three independent time points after L-glutamate stimulation were analysed. ^*^p < 0.05; ^***^p < 0.001. Statistical significance was analysed by ANOVA (Tukey test) using GraphPad software. ^$$$^*p* < 0.001 compared with 0% MG serum at 24 hours. ^##^*p* < 0.01; ^###^*p* < 0.001 compared with 0% MG serum at 48 hours. (**B**) Representative images of 3D skeletal muscle tissues incubated with MG patient sera in a dose-dependent manner. The tissues were immunostained for human membrane attack complex C5b9 and DAPI. Scale bars, 500 µm. (**C**) Bar graph indicating the level of C5b9 deposition localised on the surface of 3D skeletal muscle fibres. Data normalised to the total nuclear counts. Values are mean ± SEM. Two separate MG patient sera were assessed. ^*^p < 0.05. Statistical significance was analysed by ANOVA (Tukey test) using GraphPad software. (**D** and **E**) Biochemical analysis showed that the levels of complement split products C5a and soluble C5b9 were increased upon incubation with MG patient sera in a dose-dependent manner. Values are mean ± sd. Two independent MG patient sera were analysed. No statistical analysis was performed due to the small sample size; results are descriptive.3D, three-dimensional; AChR, acetylcholine receptor; ANOVA, analysis of variance; C5, component 5; DAPI, 4’,6-diamidino-2-phenylindole; h, hours; MG, myasthenia gravis; NMJ, neuromuscular junction; sd, standard deviation; SEM, standard error of the mean

To reveal the translational utility of the NMJ system, we examined recapitulating pathological changes in NMJ function caused by anti-AChR antibodies in sera from MG patients. Fully matured in vitro NMJ tissues were incubated with patient serum (MG-2) positive for anti-AChR antibody to evaluate its impact on neuromuscular transmission and complement activity. All NMJ tissues were supplemented with 5% normal human serum as a fresh complement source. Neuromuscular transmissions were measured from the same tissue before and after exposure with patient serum. The results revealed a significant reduction in calcium transient activity of 24.38 ± 0.34% after 24 hours and 34.68 ± 0.41% after 48 hours, following incubation with 5% MG patient serum (Fig. 3A). Notably, the inhibitory effect on neuromuscular transmission was most pronounced at a 10% serum concentration and reached saturation within 24 hours (91.67 ± 0.35%) (Fig. 3A). Localised deposition of complement C5b9 was observed on the surface of 3D skeletal muscle tissue as demonstrated by immunocytochemical analysis, reflecting that complement activation was triggered by MG patient serum (Figs. 3B and C). Additionally, two soluble complement split products – C5a (Fig. 3D) and sC5b9 (Fig. 3E) – were detected in the supernatant of MG patient sera treated with NMJ tissues. Complement activities reached saturation at serum concentrations exceeding 10% after 24 hours of incubation, consistent with observed calcium transient changes. Consequently, we incubated NMJ tissues with MG patient sera for 24 hours in subsequent experiments. These findings suggest a trend toward complement-mediated neuromuscular impairment, but due to the small sample size (*n* = 2), no statistical inference can be made and the data should be considered descriptive.

### Zilucoplan prevented complement-induced neuromuscular damage

**Fig. 4:**
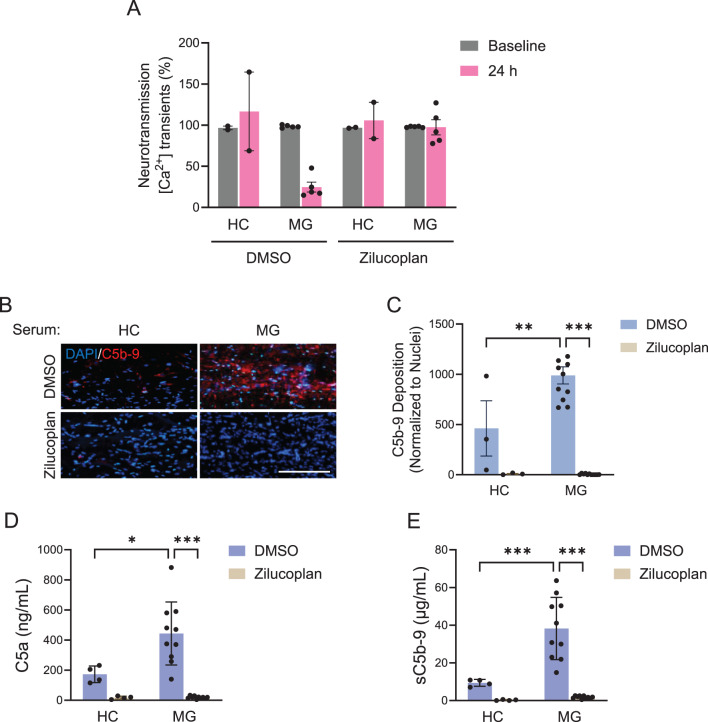
Prevention of NMJ functional impairment with zilucoplan correlates with inhibition of complement C5 on muscle cells. (**A**) incubation of the NMJ model with MG patient sera resulted in reduced neuromuscular transmission as compared to healthy control, but zilucoplan (1 µM) prevented neurotransmission impairment by MG patient sera. DMSO was used as a vehicle control for zilucoplan. Fluorescence intensity was shown as mean ± SEM. Two individual healthy control sera and five individual MG patient sera were analysed. No statistical analysis was performed due to the limited number of samples, and further studies with larger cohorts are needed to confirm these trends. (**B**) Representative images of 3D skeletal muscle tissues incubated with healthy donor and MG patient sera in the presence or absence of zilucoplan (1 µM). The tissues were immunostained for human membrane attack complex C5b9 and DAPI. Scale bars, 500 µm. (**C**) Bar graph indicating the level of C5b9 deposition localised on the surface of 3D skeletal muscle fibres. Data normalised to the total nuclear counts. Values are mean ± SEM. Three independent NMJ tissues from healthy control sera and 11 independent NMJ tissues from MG patient sera were analysed. ^**^p < 0.01; ^***^p < 0.001. Statistical significance was analysed by ANOVA (Tukey test) using GraphPad software. (**D** and **E**) Biochemical analysis showed that complement split products C5a and soluble C5b9 were indeed generated upon incubation with MG patient sera as compared to the healthy control. Furthermore, zilucoplan (1 µM) prevented the generation of C5a and soluble C5b9 induced by MG patient sera. Values are mean ± sd. Four independent samples were analysed from healthy control sera and 10 independent samples were analysed from MG patient sera. ^*^p < 0.05; ^***^p < 0.001. Statistical significance was analysed by ANOVA (Tukey test) using GraphPad software. 3D, three-dimensional; ANOVA, analysis of variance; C5, component 5; DAPI, 4’,6-diamidino-2-phenylindole; DMSO, dimethyl sulfoxide; h, hours; HC, healthy control; MG, myasthenia gravis; NMJ, neuromuscular junction; SD, standard deviation; SEM, standard error of the mean

NMJ tissues were incubated in vitro with five different anti-AChR antibody-positive MG sera to assess their impact on neuromuscular transmission and the level of complement activation in the absence or presence of a complement C5 inhibitor (zilucoplan). Zilucoplan was used at a final concentration of 1 µM in all treatment conditions. Incubation of the NMJ tissues with MG patient sera for 24 hours resulted in an 81.3% ± 9.95% reduction in neuromuscular transmission, compared with sera from healthy individuals (Fig. 4A, Supplementary Video 1). However, neuromuscular transmission was completely preserved in the presence of zilucoplan (Fig. 4A, Supplementary Video 2). MAC deposition (Figs. 4B and C and Supplementary Figure 3) and soluble complement split products, C5a (Fig. 4D) and sC5b9 (Fig. 4E), were detected from the 3D skeletal muscle tissues treated with MG sera. Zilucoplan demonstrated potent inhibition of MAC deposition on the muscle tissues and significant reductions in C5a and sC5b9 production consistent with a mode of action by which zilucoplan binds C5 and blocks its cleavage. These collective results suggest that MAC-mediated damage is a major driver of NMJ functional impairment induced by the MG patient sera tested in this study.

### Reversibility assessment of NMJ functional impairment from complement damage

**Fig. 5:**
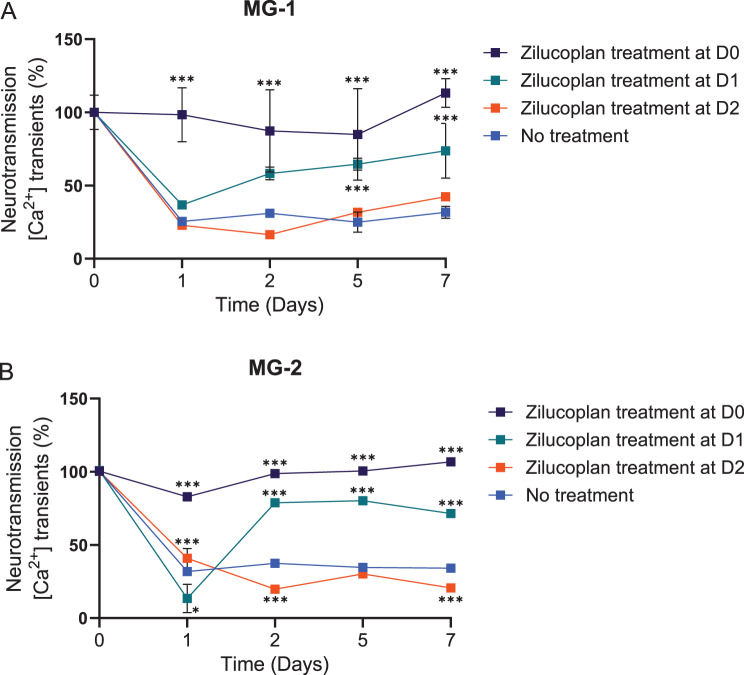
Evaluating the reversibility of NMJ functional impairment caused by MG patient sera. The in vitro NMJ model was used to evaluate the impact of complement inhibition on neuromuscular transmission when exposed to MG patient sera. NMJ tissues were incubated with (**A**) MG-1 and (**B**) MG-2 sera for either 24 or 48 hours, followed by treatment with the complement inhibitor zilucoplan for up to 7 days. Measurements of neuromuscular transmission were taken at multiple time points (Days 0, 1, 2, 5, and 7). 1 µM zilucoplan was used for all treatment groups. Fluorescence intensity was shown as mean ± sd. *n* = 3. ^*^p < 0.05; ^***^p < 0.001. Statistical significance compared with no treatment group was analysed by ANOVA (Tukey test) using GraphPad software. ANOVA, analysis of variance; D, Day; MG, myasthenia gravis; NMJ, neuromuscular junction; SD, standard deviation

While the in vitro NMJ model appeared to be well suited to providing the value of complement inhibition in preventive treatment, we further explored the relevance of the model for studies into the reversibility of NMJ damage. The NMJ tissues functionally assembled inside the NeuroMuscle^TM^ platform enabled the performance of repeated precise measurements of neuromuscular transmission from the same tissue before exposure (Day 0) and after incubation with sera from patients with MG (Days 1, 2, 5, and 7). MG patient sera induced 70–80% of functional impairment in the NMJ neurotransmission after 24 hours, which did not recover by Day 7. However, after simultaneous treatment with zilucoplan and both MG-1 and MG-2 sera (Table 1), the stability of the NMJ was maintained for up to 7 days and neurotransmission did not decline over time (zilucoplan treatment at Day 0, Figs. 5A and B). When the MG patient serum was supplemented with zilucoplan on Day 1, NMJ function was significantly recovered by Day 7. In contrast, when zilucoplan was added after 2 days of incubation with MG patient sera, no recovery in neurotransmission was observed by Day 7. These results imply that reducing the time of delay for complement inhibitor treatment in the in vitro NMJ tissues leads to improvement of the reversibility of neuromuscular transmission.

## Discussion

Here we describe an in vitro model of the human NMJ, functionally assembled inside a microfluidic device (NeuroMuscle^TM^ platform) that is compatible with standard microscope analysis. When incubated with sera from patients with MG, positive for anti-AChR autoantibodies, the NMJ developed in the NeuroMuscle^TM^ platform effectively recapitulates complement-mediated damage observed in MG. This is evidenced by the significant reduction in calcium transients following exposure to the sera. The platform revealed complement activation through the deposition of C5b9 on the surface of 3D skeletal muscle tissues and the presence of soluble complement split products, such as C5a and sC5b9, in the supernatant. These pathological changes are directly correlated with impaired neuromuscular transmission, as indicated by the decreased calcium transient activity. The ability to observe these changes in real time provides a robust 3D in vitro platform for studying the mechanistic pathways of complement-mediated damage at the NMJ.

The Phase 3 clinical trial data for zilucoplan demonstrated significant efficacy in treating MG, with improvements in key MG-specific outcomes and a favourable safety profile [14, 33]. The in vitro NMJ model supports these clinical findings by demonstrating that complement inhibition with zilucoplan preserves neuromuscular transmission and prevents complement-mediated damage. While the model does not elucidate the precise molecular mechanism of action, it effectively captures the downstream effects of complement inhibition. Conversely, the clinical efficacy of zilucoplan reinforces the relevance and accuracy of this NMJ tissue produced in vitro in mimicking the in vivo pathophysiological processes of MG. This bidirectional support underscores the model’s utility in both basic research and the evaluation of therapeutic interventions.

While the NMJ model successfully mimics several key aspects of NMJ function and pathology, it is critical to understand the domain of validity when deciding how to apply this platform for the drug development process. The platform’s ability to estimate the behaviour of complement inhibitors, such as zilucoplan, highlights its potential for drug discovery and therapeutic testing across a range of complement-driven neuromuscular disorders. This adaptability underscores the platform’s significance in advancing our understanding of NMJ pathophysiology and developing targeted treatments. Additionally, the NMJ platform could be used to assess the behaviour of other complement inhibitors, including those targeting the upper classical complement pathway. This broadens the scope of the model, allowing for comprehensive evaluation of various therapeutic strategies aimed at mitigating complement-mediated damage.

In addition to its application in studying complement-mediated damage, the platform offers a unique opportunity to explore the broader implications of immune-mediated neuromuscular disorders. By integrating various patient-derived components, such as immune cells or sera, the model could be tailored to investigate the complex interplay between the immune system and neuromuscular function. This approach could provide valuable insights into the role of immune cells, cytokines, and other inflammatory mediators in the pathogenesis of NMJ diseases. While the current model recapitulates key features of the NMJ, it does not include perisynaptic Schwann cells (PSCs), which are known to contribute to synaptic maintenance and repair. Future iterations of the model could incorporate PSCs to more fully mimic the cellular complexity of the NMJ microenvironment. Furthermore, by generating muscle fibres or MNs from patient-derived iPSCs or primary cells, the platform’s ability to incorporate genetic modifications allows for the study of specific gene mutations associated with congenital myasthenic syndromes, providing a deeper understanding of the genetic underpinnings of these disorders.

The NeuroMuscle^TM^ platform enabled quantitative analysis of the effects of postsynaptic blockers (Fig. 2C) and pathogenic autoantibodies (Fig. 3) on NMJ tissue function, as well as supported drug development (Fig. 4) and elucidation of disease mechanism of action (Fig. 4) by assessing neuromuscular transmission through calcium transients. Future advancements in the NMJ model should include additional multiparametric reading capabilities, such as quantification of the mechanical aspects of muscle contraction, for deeper understanding of the full spectrum of neuromuscular disorders and the impact of neuromuscular diseases on muscle strength and endurance [27–29]. Additionally, the ability to keep the in vitro NMJ tissue alive for multiple weeks could enable the study of the long-term effects of chronic exposure to pathogenic antibodies or the potential for muscle regeneration. Although the timing in the model does not mimic real-life situations, these factors are crucial in the progression and treatment of MG, as chronic exposure can lead to sustained damage and the body’s regenerative capacity plays a significant role in disease outcomes [34]. These improvements to the NMJ in vitro model could significantly expand its applications and the ability to fully mimic the chronic nature of NMJ disorders and the dynamic processes involved in muscle repair and regeneration.

The intricate process of fabricating microfluidic devices and the time-intensive nature of culturing and differentiating human iPSC-derived MNs and skeletal muscle fibres limit the number of experiments that can be conducted simultaneously. This constraint can hinder looking at patient heterogeneity across large numbers of patient sera, large-scale screening efforts and the rapid evaluation of multiple conditions or treatments. A high throughput version of the NeuroMuscle^TM^ platform, enabling simultaneous testing of multiple NMJ tissues would significantly accelerate drug screening, enabling mechanistic studies and the assessment of various therapeutic interventions.

Furthermore, the NMJ model can be adapted to investigate the pathogenic mechanisms of other neuromuscular diseases by incorporating patient-specific sera or autoantibodies. Thus, the versatility of this in vitro model of the human NMJ extends beyond MG, making it a valuable tool for studying other complement-driven NMJ diseases, such as Lambert-Eaton myasthenic syndrome and certain forms of congenital myasthenic syndromes.

## Conclusions

Our investigation delineates a robust and innovative approach to NMJ modelling through the application of the NeuroMuscle^TM^ microfluidic platform. This system has demonstrated exceptional proficiency in recapitulating the intricate architecture and physiology of human NMJs by fostering the coalescence of human iPSC-derived MN spheroids and 3D skeletal muscle fibres to establish functional synaptic interfaces. The fidelity of this model was further substantiated by its responsiveness to pathological sera from patients with MG, which elicited complement-mediated impairments analogous to those observed in vivo. Notably, the administration of the complement C5 inhibitor, zilucoplan, mitigated these detriments, thereby supporting its therapeutic potential as described in Phase 3 clinical trials. These findings not only validate the NeuroMuscle^TM^ platform as a potent surrogate for in vivo NMJs but also underscore its translational capacity for drug screening and mechanistic studies of other neuromuscular disorders.

## Electronic supplementary material

Below is the link to the electronic supplementary material.

Supplementary material 1

## Data Availability

The datasets presented in this article are not readily available because data from non-clinical studies are outside of UCB’s data-sharing policy and are unavailable for sharing.

## Abbreviations

3D: Three-dimensional
ACh: Acetylcholine
AChR: Acetylcholine receptor
ANOVA: Analysis of variance
BTX: α-bungarotoxin
C5: Component 5
D: Day
DAPI: 4’,6-diamidino-2-phenylindole
DMEM: Dulbecco’s modified eagle medium
DMSO: Dimethyl sulfoxide
DTC: d-tubocurarine
EMG: Electromyography
GCaMP6: Genetically encoded calcium indicator protein 6
h: Hours
HC: Healthy control
hESC: Human embryonic stem cell
IGF-1: Insulin-like growth factor 1
iPSC: Induced pluripotent stem cell
MAC: Membrane attack complex
MEM: Minimum essential medium
MG: Myasthenia gravis
MN: Motor neuron
N/A: Not applicable
NMJ: Neuromuscular junction
PBS: Phosphate-buffered saline
PDMS: Polydimethylsiloxane
PSC: Perisynaptic Schwann cell
RT: Room temperature
SAA: Serum amyloid A
SD: Standard deviation
SEM: Standard error of the mean
Tuj1: Class III beta-tubulin
VGLUT1: Vesicular glutamate transporter 1
