# Supplementary material for: Human in vitro neuromuscular junction model to functionally dissect the pathogenic mechanism of anti-AChR autoantibody-positive myasthenia gravis
Source: BMC Pharmacol Toxicol. 2025 Dec 12;27:17. doi: 10.1186/s40360-025-01056-1 (PMC12817423; doi:10.1186/s40360-025-01056-1)
Supplement: Supplementary file 1 — Supplementary material 1 [file 40360_2025_1056_MOESM1_ESM.pptx]

## Slide 1
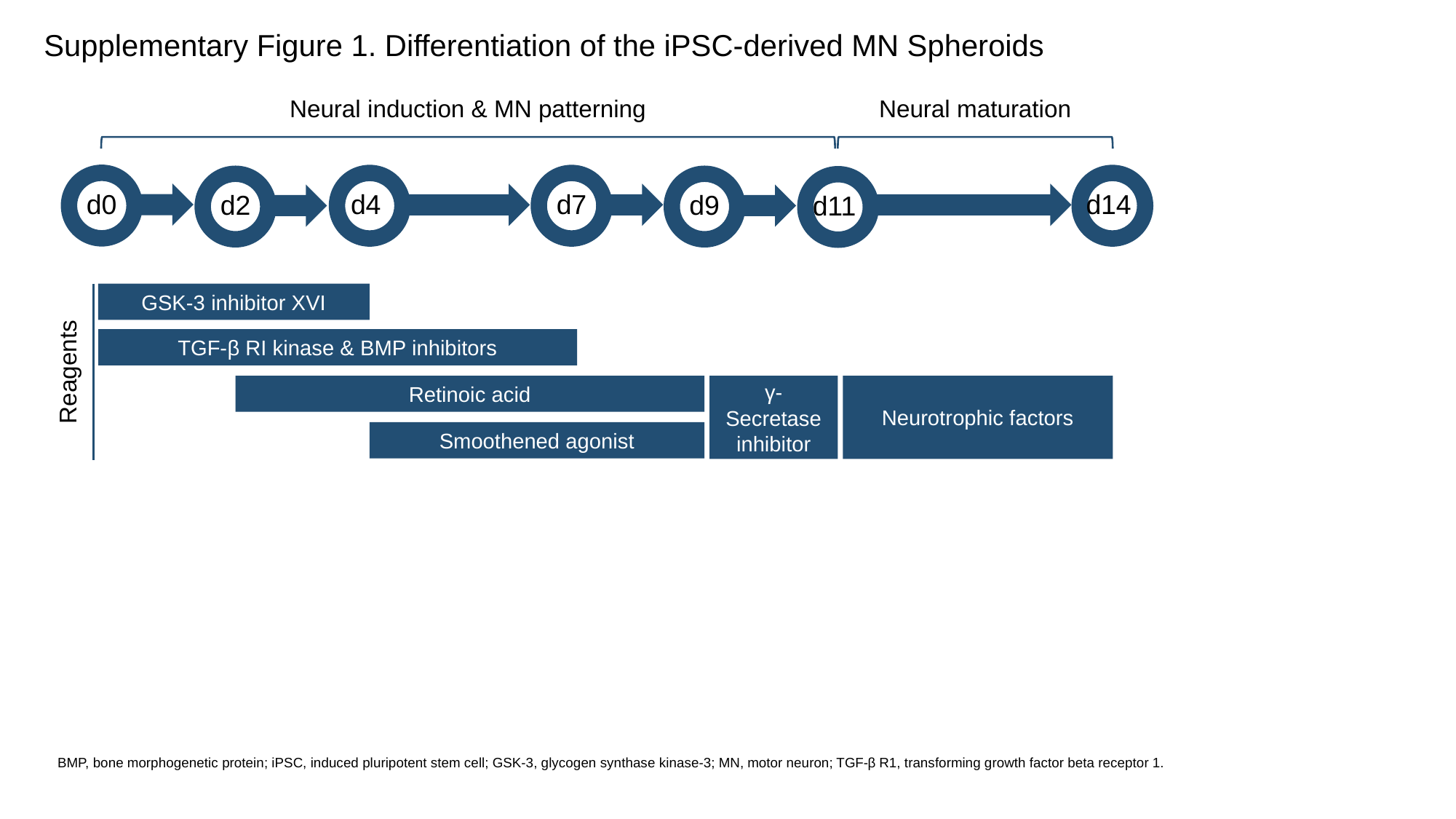

Supplementary Figure 1. Differentiation of the iPSC-derived MN Spheroids
Neural induction & MN patterning
Neural maturation
d0
d4
d7
d14
d2
d9
d11
GSK-3 inhibitor XVI
TGF-β RI kinase & BMP inhibitors
Reagents
γ-Secretase inhibitor
Neurotrophic factors
Retinoic acid
Smoothened agonist
BMP, bone morphogenetic protein; iPSC, induced pluripotent stem cell; GSK-3, glycogen synthase kinase-3; MN, motor neuron; TGF-β R1, transforming growth factor beta receptor 1.

## Slide 2
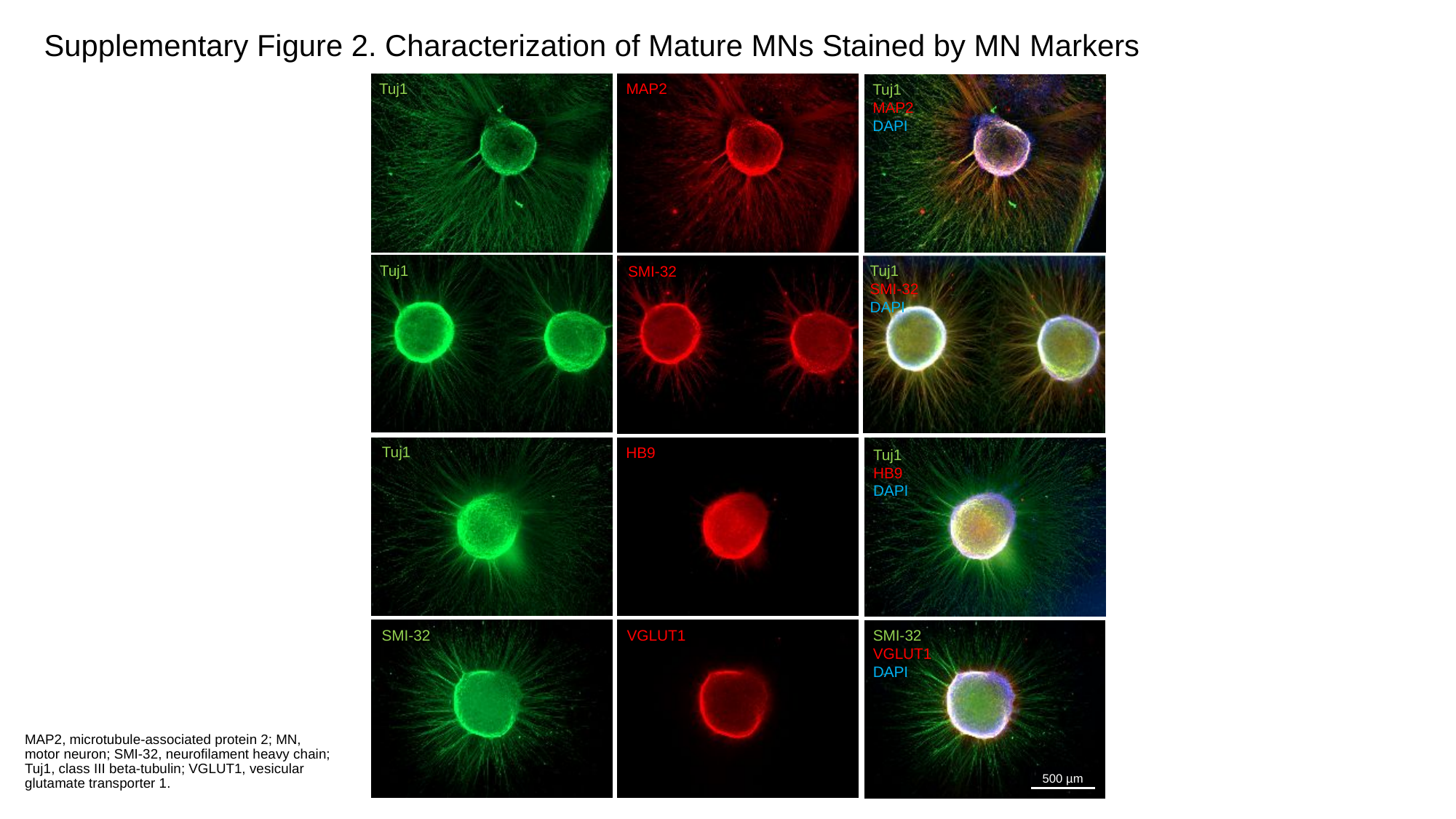

Supplementary Figure 2. Characterization of Mature MNs Stained by MN Markers
Tuj1
MAP2
Tuj1
MAP2
DAPI
Tuj1
Tuj1
SMI-32
DAPI
SMI-32
Tuj1
HB9
Tuj1
HB9
DAPI
VGLUT1
SMI-32
VGLUT1
DAPI
SMI-32
MAP2, microtubule-associated protein 2; MN, motor neuron; SMI-32, neurofilament heavy chain; Tuj1, class III beta-tubulin; VGLUT1, vesicular glutamate transporter 1.
500 µm

## Slide 3
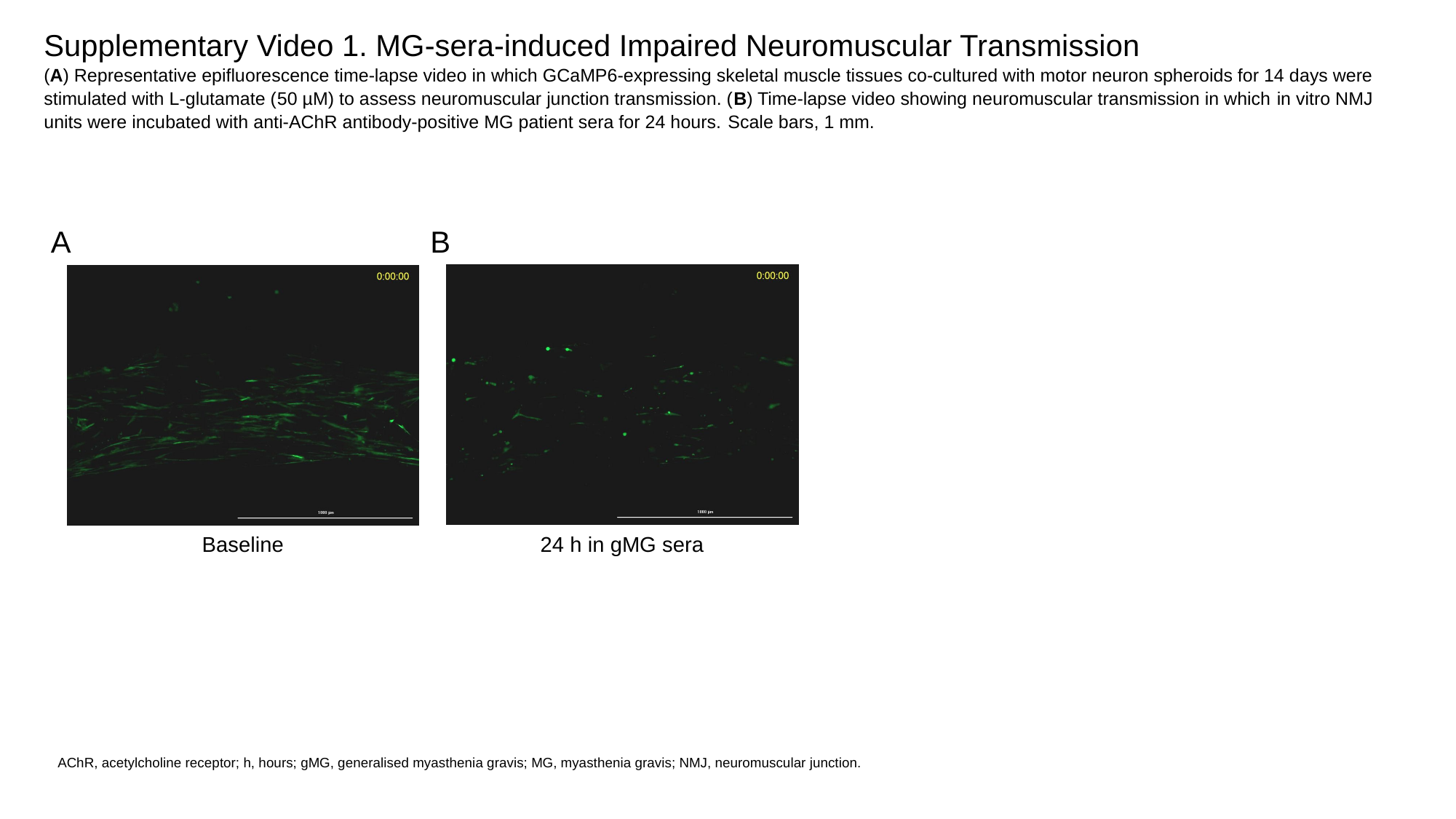

Supplementary Video 1. MG-sera-induced Impaired Neuromuscular Transmission
(A) Representative epifluorescence time-lapse video in which GCaMP6-expressing skeletal muscle tissues co-cultured with motor neuron spheroids for 14 days were stimulated with L-glutamate (50 µM) to assess neuromuscular junction transmission. (B) Time-lapse video showing neuromuscular transmission in which in vitro NMJ units were incubated with anti-AChR antibody-positive MG patient sera for 24 hours. Scale bars, 1 mm.
A
B
Baseline
24 h in gMG sera
AChR, acetylcholine receptor; h, hours; gMG, generalised myasthenia gravis; MG, myasthenia gravis; NMJ, neuromuscular junction.

## Slide 4
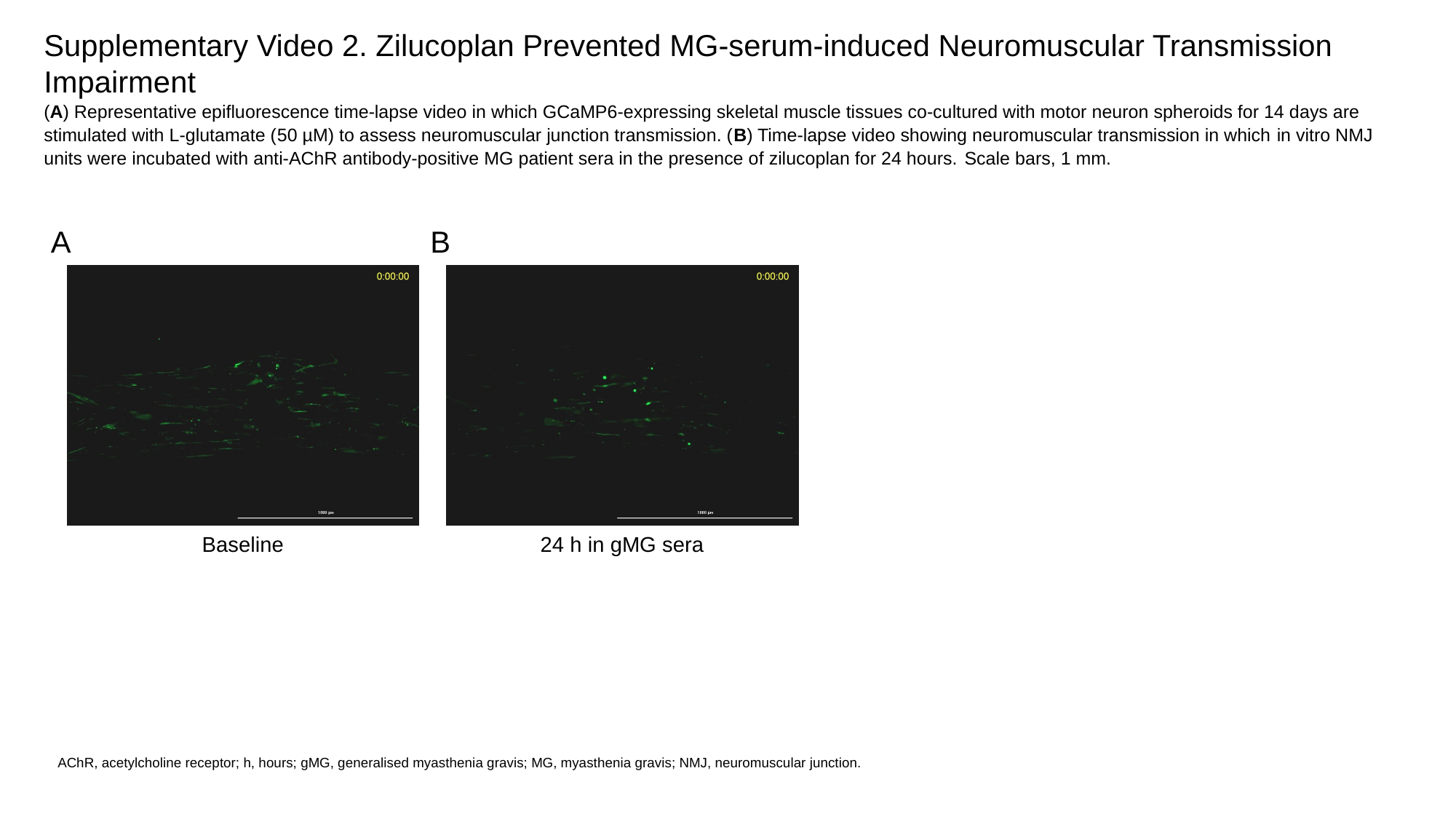

Supplementary Video 2. Zilucoplan Prevented MG-serum-induced Neuromuscular Transmission Impairment
(A) Representative epifluorescence time-lapse video in which GCaMP6-expressing skeletal muscle tissues co-cultured with motor neuron spheroids for 14 days are stimulated with L-glutamate (50 µM) to assess neuromuscular junction transmission. (B) Time-lapse video showing neuromuscular transmission in which in vitro NMJ units were incubated with anti-AChR antibody-positive MG patient sera in the presence of zilucoplan for 24 hours. Scale bars, 1 mm.
A
B
Baseline
24 h in gMG sera
AChR, acetylcholine receptor; h, hours; gMG, generalised myasthenia gravis; MG, myasthenia gravis; NMJ, neuromuscular junction.

## Slide 5
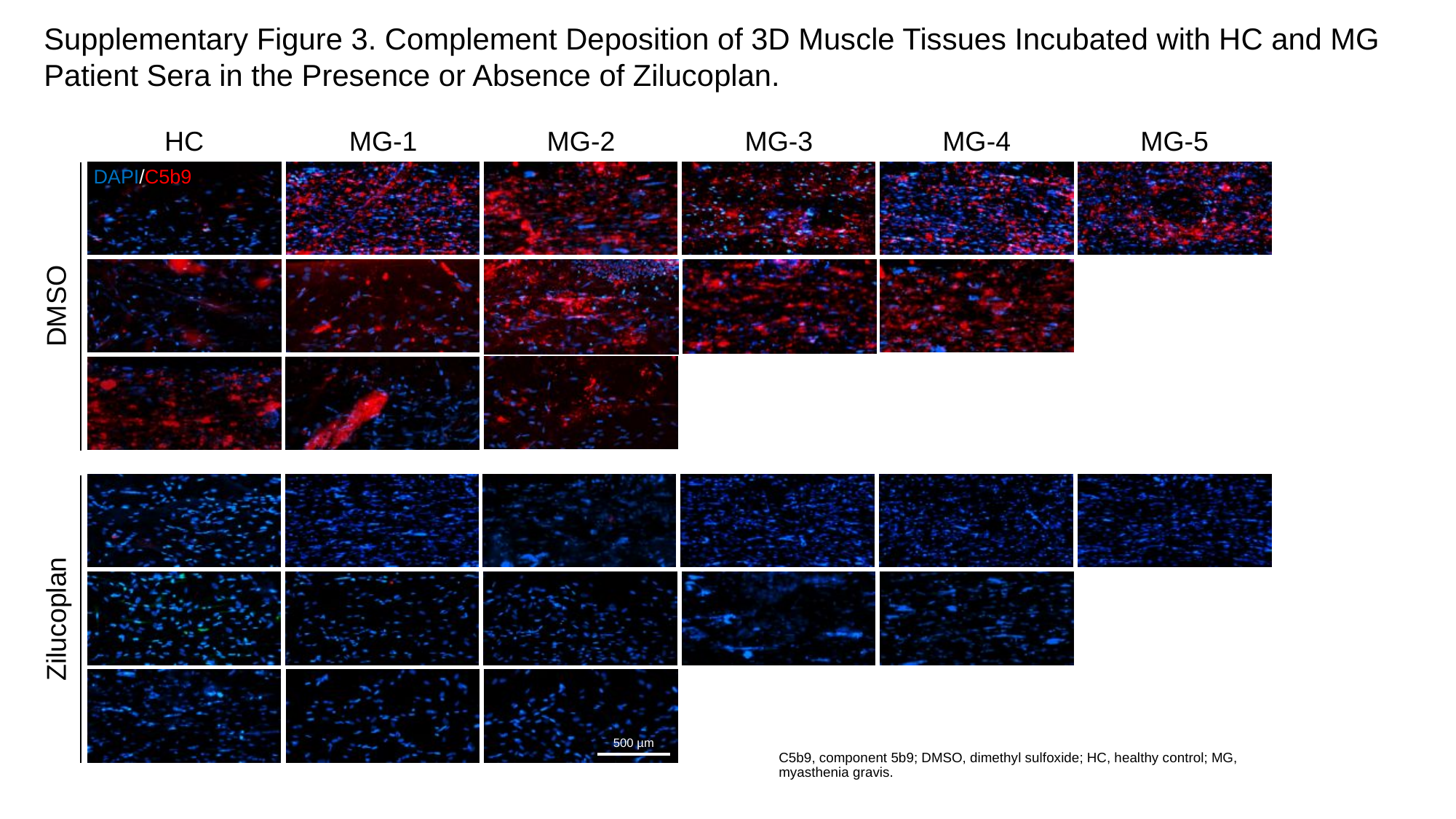

Supplementary Figure 3. Complement Deposition of 3D Muscle Tissues Incubated with HC and MG Patient Sera in the Presence or Absence of Zilucoplan.
HC
MG-1
MG-2
MG-3
MG-4
MG-5
DAPI/C5b9
DMSO
Zilucoplan
500 µm
C5b9, component 5b9; DMSO, dimethyl sulfoxide; HC, healthy control; MG, myasthenia gravis.
